# Comparative proteomics reveals different protein expression in platelets in patients with alcoholic liver cirrhosis

**DOI:** 10.1186/s12953-024-00227-y

**Published:** 2024-01-26

**Authors:** Nima Haji Begli, Cora Freund, Karl-Heinz Weiss, Daniel Gotthardt, Andreas Wannhoff

**Affiliations:** 1 Department of Internal Medicine IV, University Hospital Heidelberg, Im Neuenheimer Feld 410, 69120 Heidelberg, Germany; 2 Salem Medical Center, Zeppelinstr. 11-33, 69120 Heidelberg, Germany; 3 Department of Internal Medicine and Gastroenterology, Hospital Ludwigsburg, Posilipostrasse 4, 71640 Ludwigsburg, Germany

**Keywords:** Platelet proteomics, Alcoholic liver cirrhosis, Rab-7a, RANBP1, RhoGDI1, 14–3-3 gamma

## Abstract

**Background:**

The role of platelets in disease progression as well as the function of platelets as part of the haemostatic and immunological system in patients with liver cirrhosis is only incompletely understood. This is partly due to difficulties in assessing platelet function. Proteome analyses of platelets have been used to further investigate the role of platelets in other diseases.

**Aim:**

To assess possible changes in the platelet proteome during different stages of alcohol induced liver cirrhosis compared to healthy donors.

**Patients and methods:**

A 45 ml blood sample was drawn from 18 participants aged 18–80 years evenly divided into three groups of healthy donors, patients with less advanced alcohol induced liver cirrhosis (Child-Pugh < 7) and patients with advanced liver cirrhosis (Child-Pugh > 10). The blood was processed to isolate platelets and perform subsequent two-dimensional gel-electrophoresis using a SYPRO™ Ruby dye. After computational analysation significantly in- or decreased protein spots (defined as a two-fold abundance change between different study cohorts and ANOVA < 0.05) were identified via liquid chromatography–mass spectrometry (LCMS) and searching against human protein databases.

**Results:**

The comparative analysis identified four platelet proteins with progressively decreased protein expression in patients with liver cirrhosis. More specifically Ras-related protein Rab-7a (Rab-7a), Ran-specific binding protein 1 (RANBP1), Rho GDP-dissociation inhibitor 1 (RhoGDI1), and 14–3-3 gamma.

**Conclusion:**

There is significant change in protein expression in the platelet proteome throughout the disease progression of alcohol induced liver cirrhosis. The identified proteins are possibly involved in haemostatic and immunoregulatory function of platelets.

**Supplementary Information:**

The online version contains supplementary material available at 10.1186/s12953-024-00227-y.

## Introduction

Chronic liver disease and especially liver cirrhosis is usually accompanied by multiple changes within the haemostatic system. These include abnormalities of multiple coagulation factors affecting pro- and anticoagulant factors as well as the fibrinolytic system, thrombocytopenia and potentially thrombocytopathy [1, 2]. Conventional coagulation tests typically show an increased international normalized ratio (INR) and a reduced platelet count and thus patients with liver cirrhosis were long believed to have an acquired coagulation disorder with increased risk of bleeding and were thought to be naturally anticoagulated [3]. It is nowadays no longer believed that patients with liver cirrhosis are “naturally” anticoagulated but rather have a re-balanced haemostatic system that might lead to bleeding or thrombosis through changes in pro- and anticoagulant factors [4] and therefore go from a healthy balanced state to a re-balanced state throughout their disease progression. There also is evidence that suggests increased coagulation in patients with cirrhosis and these patients do show an increased risk of peripheral thrombosis [5]. While the coagulation system is well studied in liver cirrhosis and thrombocytopenia is known to be a hallmark of end-stage liver disease (ESLD), less is known about platelet function and the presence of thrombocytopathy [6].

Furthermore, research in recent years has increasingly shown that platelets not only play a primary role in haemostasis as described above, but also participate in the immune response, the resolution of inflammation [7, 8] and angiogenesis [9]. In addition, recent data shows that in liver fibrosis, platelets are involved in the development of hepatic fibrosis via complex interactions with hepatic stellate cells (HSC) [10, 11]. Interestingly both activating and inhibiting HSC are consequently leading to hepatic fibrosis through a continuous injury response.

Thus, it is of high interest to shed a light on possible changes in platelet function in patients with liver cirrhosis; yet measurement of platelet function and activity remains challenging. Investigation of the platelet proteome by means of Proteomics in various diseases has been shown to be possible and promising [12, 13].

We hypothesized that there is presence of thrombocytopathy in patients with ESLD and aimed to investigate this by performing analyses of the platelet proteome in patients with alcohol induced liver cirrhosis and healthy controls.

## Materials and methods

### Study protocol

Platelet analysis was planned in patients with alcoholic liver cirrhosis as well as in a control cohort (Group Contr) of participants without liver disease. The group of patients with cirrhosis was planned to be split in two subgroups with less advanced disease (Group Cirr1; defined as Child Pugh score ≤ 7) and with advanced disease (Group Cirr2; Child Pugh score > 10). A total of 18 participants, six in each of the three groups, were planned to be included. Exclusion criteria were as followed: (1) platelet count < 50.000, (2) diagnosis of thrombotic diseases, (3) use of antithrombotic medication in the last 4 weeks or scheduled to start of such medication and/or history of anticoagulation, (4) malignant disease – patients were as well excluded per protocol during a 12 month follow up period if there was a new cancer diagnosis, and (5) relapse in alcohol consumption in the last 4 months prior to enrolment – in order to exclude acute myelosuppressive changes of alcohol consumption. Patients treated at Department of Internal Medicine IV of University Hospital Heidelberg were screened for inclusion. Six healthy donors were paired by age and sex and were enrolled in the study as controls.

The study was previously approved by the ethics committee of the University of Heidelberg and all participants provided written informed consent prior to inclusion in the study.

### Plasma collection, platelet separation and storage

The preparation of blood samples was primarily conducted in accordance with established protocols [14, 15], with minor modifications implemented for optimized processing.

In total five vials each containing 9 ml blood sample and 1 ml sodium citrate stock solution (equalling a final concentration of 10% (v/v) with anticoagulant) were drawn as usual for the control and patient group. Each blood sample was processed individually. One ml citrate dextrose solution (ACD) stock solution was added to each vial prior to centrifugation at 200×g (times gravity) for 20 min. The upper third of the platelet rich plasma (PRP) was transferred to a new tube to avoid contamination with leukocytes and Prostacyclin (2.5 mM) was added to avoid platelet activation. The sample was centrifuged at 1000×g for 10 min. The resulting pellet was washed with modified Tyrodes-HEPES-buffer ((134 mM NaCl, 0.34 mM Na_2_HPO_4_, 2.9 mM KCl, 12 mM NaHCO_3_, 20 mM HEPES, 5 mM glucose, 1 mM MgCl_2_, 1 mM EGTA, 10 M indomethacin, ACD (7%, v/v), pH 7.3) and again centrifuged at 1000 g for 10 minutes. Then, platelets were resuspended at a concentration of 2 × 10^8^ cells/ml with Tyrodes-HEPES buffer, incubated for 30 minutes at room temperature, and centrifuged at 10000×g for 2 minutes. Finally, protease and phosphatase inhibitor (Halt™ Protease and Phosphatase Inhibitor Cocktail, Thermo Fisher Scientific, Waltham, USA) were added and resulting pellets were stored at − 80 °C for further analysis.

### Cell lysis and protein determination

Prior to further analysis each sample was thawed on iced. The pellets were then suspended in 100 μl 0.9% NaCl and spun for 2 minutes. Following, each pellet was added to 50 μl TCA and resuspended with 350 ml lysis buffer ((9.5 M Urea, 4% CHAPS, 65 mM DTT, 0.2% carrier ampholyte, and 20 μl/ml protease inhibitor cocktail). Subsequently, the lysed platelets were subjected to centrifugation at a speed of 14,000 rpm for 60 minutes. The supernatant, enriched in soluble proteins, was then employed for 2-dimensional gel electrophoresis. The protein concentration of each sample was quantified using the Bradford protein assay.

### Isoelectric focusing

The first step of isoelectric focusing (IEF) was done using immobilized pH gradient (IPG) strips (non-linear gradient, 18 cm, pH 3–10, Thermo Fisher Scientific, Waltham, USA). Each strip was rehydrated for 5 h at 30 V, 6 h at 60 V and 2 h at 100 V. IEF was conducted at 200 V, 500 V and 1000 V for each 1,5 h followed by a gradient from 1000 V to 8000 V and focusing at 8000 V for 8 h to reach a total of approx. 60–80 kV. After completion of IEF, the IPG strips were immediately used for further analysis.

### 2-DE analysis

Equilibration was performed via incubation of the strips in equilibration buffer for 20 minutes with gentle shaking (1% w/v DTT, 50 mM, pH 6.8 Tris–HCl, 6 M Urea, 30% Glycerol, 2% SDS, bromophenol blue) followed by a second 20 min equilibration in (2.5% w/v IAA instead of DTT). The strips were then added for separation in the second dimension (molecular weight) to 12.5% acrylamide gels. Bromphenol blue was added to visualize the progress of the separation process. PageRuler Plus Prestain Protein (10 to 250 kDa) was used as a protein marker. The separation was done as soon as the blue front reached the bottom. Following two fixation steps of each 30 minutes by placing the gels in a 50% methanol and 7% acetic acid solution visualization was done by SYPRO™ Ruby (Thermo Fisher Scientific, Waltham, USA) protein gel stain following the staining method recommended by the manufacturer. Each sample was run in both a technical and biological duplicate to ensure reproducibility.

### Scan of the 2-DE gels

The scanning of each gel was performed using a Molecular Imager® FX scanner (Bio-Rad Laboratories, California, USA). Following the manufacturer’s instructions, the light source was set to a wavelength of 488 nm for the analysis of the 2-DE gels, to optimally excite the fluorescent dyes or labeled proteins. The emission filter was calibrated to 640 ± 35 nm to precisely isolate the light emitted by the dyes. These specific settings ensure that only the desired emitted light is captured, thereby minimizing interference signals and enabling a clear and accurate representation of the protein spots on the gel. The resolution of each scan was 530 pixels per millimeter.

#### Image acquisition and data analysis

The image analysis was conducted with Image Master 2 (General Electric, Boston, USA). The resolution was 530 pixels per millimetre. Several parameters were fixed to ensure comparable data for quantitative analysis. Each subgroup (controls, mild and advanced liver cirrhosis) was grouped creating a reference gel for further analysis following the instructions of the developer. To generate the reference gels for further analysis, the digital data of the IPG strips within each subgroup was pooled using the Image Master software. This digital pooling process allowed us to create a representative representation of the overall protein patterns for each subgroup. Grouping was done according to the proprietary parameters pre-set in the program. Internal software filters excluded experimental artifacts. Furthermore, each gel was resized manually to ensure best possible comparability. Spots with a 2-fold abundance change and an ANOVA < 0.05 were accepted as statistically significant and selected for protein identification. Increased volume by 50% was defined as up-regulation and decreased volume by 50% was defined as down-regulation.

#### In-gel-digestion and peptide extraction

Protein digestion and LC-MS measurement were done as previously explained elsewhere [16]. In short, protein spots of interest were excised manually from the reference gels. Following a reduction with 40 mM dithiothreitol and alkylation with 50 mM iodoacetamide gel pieces were dehydrated. Trypsin solution (sequencing grade, Thermo-Fisher, Rockford, USA) was added to the dry gel pieces and incubated over night at 37 degrees Celsius. The reaction was quenched by addition of 20 μL of 0.1% trifluoroacetic acid (TFA; Biosolve, Valkenswaard, Netherlands). The supernatant was dried in a vacuum concentrator before LC-MS analysis.

#### LC-MS measurements

Nanoflow LC-MS analysis was performed with NanoAcquity UPLC liquid chromatography (Waters, Eschborn, Germany) system coupled to an Orbitrap XL (Thermo-Fischer, Bremen, Germany). Samples were loaded to a NanoAcquity Symmetry C18 Trap column (particle size 5 μm, inner diameter 180 μm × 20 mm, Waters) at a flow of 15 μL/min for 5 min of 0.5% solvent B. Peptides were separated using a NanoAcquity M-Class peptide BEH C18 analytical column (particle size 1.7 μm, inner diameter 75 μm × 250 mm, Waters) with a 1 h linear gradient (3–40% B) with a flow rate of 300 nL/min. Solvent A was 0.1% formic acid (FA; ProteoChem, Denver, CO, USA) in H2O (Biosolve) and solvent B was composed of 0.1% FA (ProteoChem), 10% H2O (Biosolve) and 89.9% ACN (Biosolve). The mass spectrometer was operated in data-dependent acquisition mode, automatically switching between MS and MS 2. MS spectra (m/z 400–1600) were acquired in the Orbitrap at 60,000 (m/z 400) resolution, with an automatic gain control (AGC) target value of 5 × 10 and maximal ion injection time (IT) 50 ms. Collision induced dissociation MS 2 spectra were generated for up to 10 precursors with normalized collision energy of 35% in the ion trap. The MS 2 AGC target value was set to 10 4 with a maximum IT of 100 ms.

#### Protein identification

Raw files were analyzed using Proteome Discoverer with the Sequest (Thermo Fisher Scientific, San Jose, USA; version 2.2). Sequest was set up to search against Uniprot human databases (June 2017, https://www.uniprot.org/) with trypsin as the digestion enzyme. A fragment ion mass tolerance was set to 0.50 Da and a parent ion mass tolerance to 10 ppm. Carbamidomethyl of cysteine was specified as a fixed modification. Deamidation of asparagine and glutamine, oxidation of methionine and acetyl of the N-terminus were specified as variable modifications. The peptide and protein identity were verified by Scaffold (version Scaffold_4.8.4, Proteome Software Inc., Portland, USA). Peptide identifications were accepted if they could be established at greater than 95.0% probability by the Peptide Prophet algorithm [17] with Scaffold delta-mass correction. Protein identifications were accepted if they could be established at greater than 95.0% probability and contained at least 2 identified peptides. Protein probabilities were assigned by the Protein Prophet algorithm [18]. Proteins that contained similar peptides and could not be differentiated based on MS/MS analysis alone were grouped to satisfy the principles of parsimony.

#### Statistical analysis

All data are expressed as mean with standard deviation (SD). Statistical analysis was conducted by SPSS Version 22 (IBM Corp., Armonk, USA) as well as Image Master 2 (General Electric, Boston, USA). A *p*-value less than 0.05 was defined as statistically significant.

## Results

### Study cohort

Between March 2015 and February 2017, 18 participants were included, six in each subgroup. Participants had a mean age of 52 (range: 39–60) and 9 participants (50.0%) were male. Baseline characteristics of all three subgroups are presented in Table 1.

**Table 1:** Study cohort characteristics

	Control	Less advanced	Advanced
Sex (m/f)	3/3	3/3	3/3
Age (Range/Mean)	40–60 / 50 y	39–60 / 51.5 y	48–59 / 54.5 y
Platelet Count/nl (Range/Mean)	193–352 / 262	142–297 / 207	93–201 / 134
Red Blood Cell Count/pl (Range/Mean)	4.4–6.1 / 5.3	4.2–6 / 5.1	3.9–5.2 / 4.7
White Blood Cell Count/nl (Range/Mean)	4.3–10.3 / 8.3	3.9–12.2 / 8.2	3.1–11.4 /. 7.7

Table containing the characteristics of the study cohort. Red blood cell count and white blood cell count rounded to first decimal

### 2-DE profiles of healthy volunteers and mild/moderate liver cirrhosis patients

**Fig. 1:**
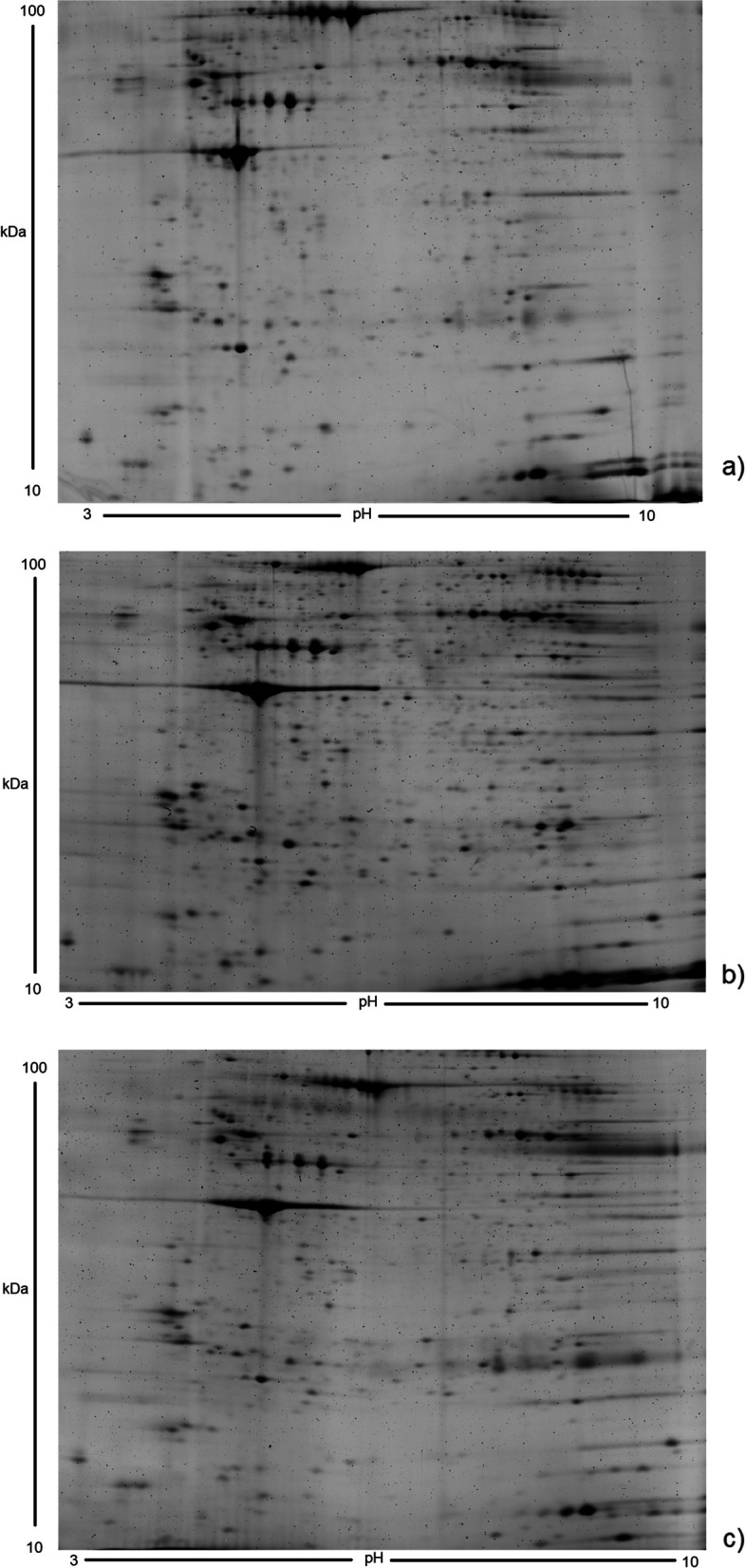
Two-dimensional reference gels. Two-dimensional reference gels of six healthy controls (**a**), six patients with less advanced alcohol induced cirrhosis (**b**) and advanced disease respectively (**c**). Each sample was run both in a technical and biological duplicate. Reference gels were obtained using the proprietary parameters preset in the analysis program. Original gels are presented in Supplementary Figs. 1, 2 and 3

The group of healthy patients was named Contr and the liver cirrhosis patients were named Cirr1 and Cirr2 respectively. Highly consistent and reproducible gel images were obtained for platelet samples of all three groups (Contr, Cirr1, and Cirr2), as shown in Fig. 1. For the platelet samples of Contr an average of 1577 spots were resolved. For the platelet samples of liver cirrhosis averages of 1752 (Cirr1) and 1355 (Cirr2) spots were resolved. In total 220 spots matched between all the groups. The correlation between technical replicates was moderate to strong, with a Pearson’s correlation coefficient ranging from 0.66 to 0.83. The correlation between biological replicates was also moderate to strong, with a Pearson’s correlation coefficient ranging from 0.58 to 0.75.

### Differential platelet 2-DE profiles between healthy volunteers and cirrhosis patients

**Fig. 2:**
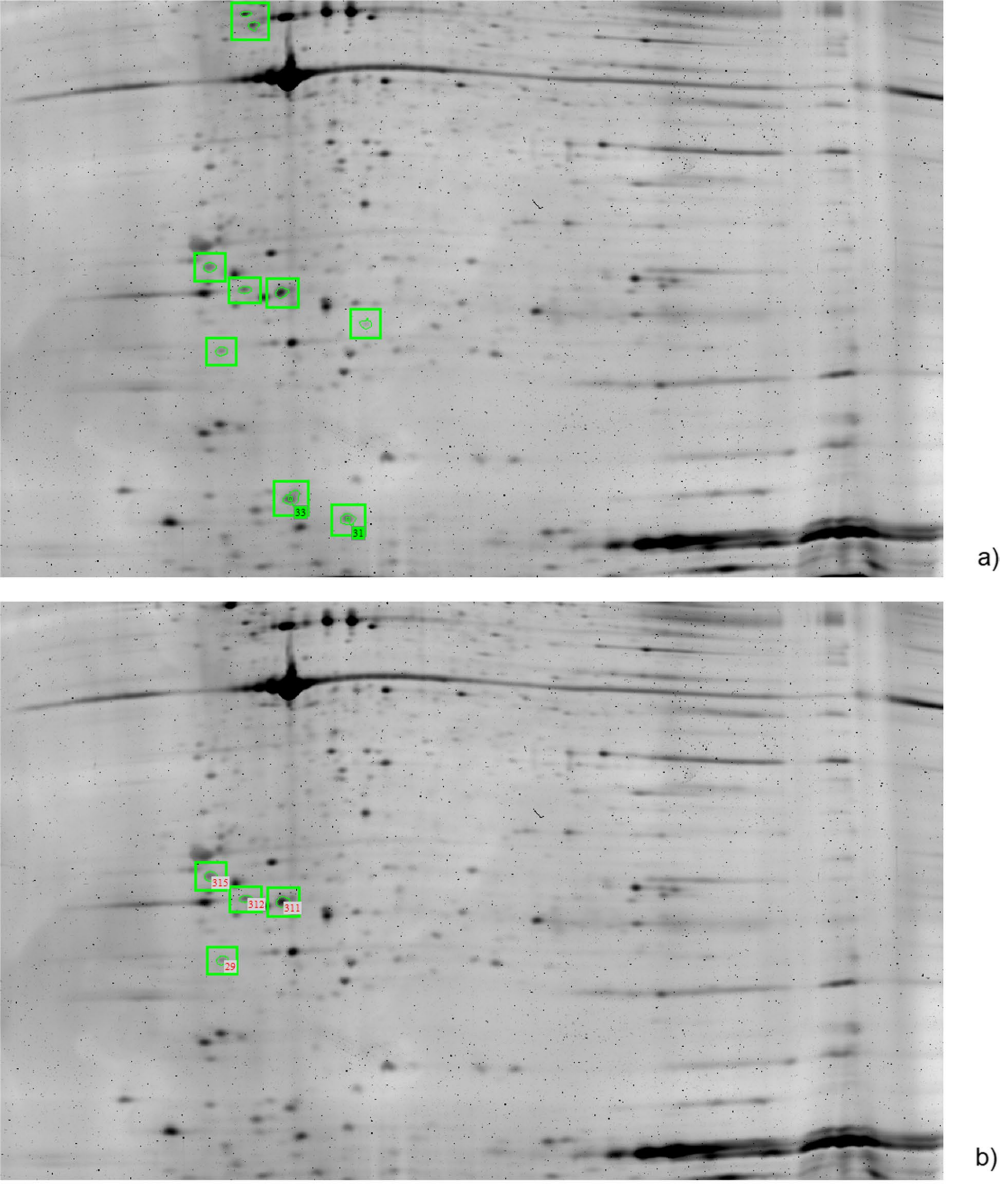
Statistically significant protein spots. Nine spots (**a**) (highlighted in green) in total fulfilled the criteria to be accepted as statistically significant (2-fold abundance change and an ANOVA < 0.05) and were selected for protein identification. Four of them (**b**) were spots without mixed protein profiles and showed platelet proteins. Original gel is presented in Supplementary Fig. 4

In total nine protein spots with varying degrees of increases of protein expression were shown to be statistically significant (*p* < 0.01). These were spots 10, 11, 29, 143, 153, 306, 311, 312 and 315. As shown in Fig. 2.

### Identification of the various 2-DE spots by mass spectrometry

These expressed and statistically significant proteins were manually excised, subjected to in-gel digestion and analysed by LC-MS as described earlier. Spots 10, 11, 143, 153 and 306 were mixed spots. In our proteomic analysis, we encountered challenges in the precise identification of proteins within certain “mixed” 2-DE gel spots. This difficulty arose due to the co-migration of multiple proteins into single spots on the gel, a phenomenon attributable to similarities in their molecular weights and isoelectric points. Consequently, mass spectrometry analysis of these spots revealed spectra indicative of several proteins, complicating the process of distinct protein identification. Such co-localization limited our ability to unambiguously attribute the observed proteomic changes to specific protein species within these mixed spots.

The remaining spots successfully showed platelet proteins which were identified as followed: Spot 29 (ras-related proteins eg. Rab 7a), spot 311 (Ran-specific GTPase-activating protein), spot 312 (Rho GDP-dissociation inhibitor 1) and spot 315 (14–3-3 gamma). All the aforementioned spots were down-regulated (2.7–10 fold decrease, *p*-value ranging from 0.001 to 0.4). The special characteristics of aforementioned spots are described in Table 2.

**Table 2:** List of proteins of significance

Spot	Name	MW gel^a^/theory^b^	*p*-value	Fold	Function
29	Ras-related prot.	20/20–24	0.01	7,3	Rab protein signal transduction
311	Ran-specific GTPase-activating protein	Approx 22/23	0.001	3	nucleocytoplasmic transport
312	Rho GDP-dissociation inhibitor 1	Approx 23/23	0.01	10	GDP/GTP exchange
315	14–3-3 gamma	Approx 27/29	0.04	2,7	Adapter protein in signaling pathways

List of proteins accepted as statistically significant showing an at least 2-fold abundance change and a *p*-value of < 0.05 ^a^Molecular weight found in gel ^b^Molecular weight found in theory.

All four proteins with significantly altered expression demonstrated a progressive decrease in protein expression, both in comparison to patients without liver cirrhosis and when transitioning from mild to severe liver cirrhosis. The detailed values and differences in protein expression of these four proteins are presented in Table 3.

**Table 3:** Listing of the protein expression of the four proteins with significantly altered expression. The measurement of the expression values was conducted using Image Master 2. The respective expression value is indicated in a unit of %Vol

Spot	Name	Protein expression (Control)	Protein expression (Less advanced)	Protein expression (Advanced)
29	Ras-related prot.	0,065	0,031	0,009
311	Ran-specific binding protein 1	0,06	0,019	< 0,01
312	Rho GDP-dissociation inhibitor 1	0,108	0,011	0,002
315	14–3-3 gamma	0,086	0,031	0,012

In total four proteins with significantly decreased protein expression were identified in the analysis between patients with liver cirrhosis and healthy donors. These down-regulated proteins were Ras-related protein 7a (Rab-7a; Protein Accession Number: P51149), Ran-specific GTPase activating protein (RANBP1; Protein Accession Number: P43487), Rho-GDP dissociation inhibitor 1 (rho GDI 1; Protein Accession Number: P52565) and 14–3-3 gamma (Protein Accession Number: P61981). Among these proteins Rab-7a belongs to the Ras-related protein family which influences the RAB protein signal transduction, RANBP1 plays a role in RAN-dependent nucleocytoplasmic transport, rho GDI 1 controls Rho proteins homeostasis and furthermore regulates the GDP/GTP exchange reaction of the Rho proteins. Finally, 14–3-3 gamma belongs to the 14–3-3 protein family implicated in the regulation of various general and specialized signalling pathways. To our knowledge all the aforementioned proteins were previously found in platelet proteomics studies.

## Discussion

In this study we conducted a comprehensive platelet proteomic analysis from patients with compensated and decompensated alcohol induced liver cirrhosis and non-cirrhotic controls.

The comparative analysis identified four platelet proteins, namely Ras-related protein Rab-7a (Rab-7a), Ran-specific binding protein 1 (RANBP1), Rho GDP-dissociation inhibitor 1 (RhoGDI1), and 14–3-3 gamma with progressively decreased protein expression in patients with liver cirrhosis. This indicates that alcoholic liver cirrhosis is associated with changes in protein expression in platelets These proteins are either directly and or indirectly linked to pathways associated with the haemostatic and possibly immunoregulatory function of platelets. As a consequence there might be altered haemostatic function of platelets in cirrhosis (i.e., thrombocytopathy) and a change in immunoregulatory function of platelets might play a role in cirrhosis progression.

Rab-7a is part of the most abundant family of small GTPases. Originally described as Ras-like proteins in the brain (Rab) these GTPases are often referred to as switches and regulators of vesicle trafficking in both endocytic and exocytic pathways. Rab-7a specifically plays a role in advanced stages of endosomal vesicle trafficking in platelets and is associated with advanced endocytic compartments and transport to lysosomes [19]. Consequently, GTPases are an important component of platelet function in both haemostasis and immunological regulation [20]. Although the precise process of Rab7a in platelet function and membrane trafficking remains unclear. Furthermore, the pathways of interaction and influence of Rab7a regarding the platelet granule secretion warrant further research. But the consequently influenced process of platelet granule secretion and the composition of its granules play a role in haemostatic and possible immunological pathways [21].

The function of RANBP1 in platelets is currently unclear but the protein itself was identified in platelets in the past [22]. It is known that RANBP1 is a Ran/TC4-binding protein and interacts especially with GTP-charged Ras-like nuclear (RAN) [23]. RAN is the most abundant intracellular small GTPase. RAN acts as a molecular switch that alternates between an active GTP-bound and an inactive GDP-bound state. As already mentioned with Rab-7a the specific pathways of regulation are unclear.

The third protein with progressively decreased protein expression Rho GDP-dissociation inhibitor 1 is one of two Rho GDI dissociation proteins (RhoGDIs) expressed in platelets [24]. These RhoGDIs are responsible for tuning and adjusting Rho GTPase activity [25]. Due to its high expression (approximately 21,000 copies per platelet) Rho GDP-dissociation inhibitor 1 provides a plausible candidate for the inhibitory regulation of Rho GTPases [24, 26]. These GTPases are part of the Rho family and are small signalling G proteins and a subfamily of the Ras (Rat sarcoma virus) superfamily. In the last decades members of the RhoGTPase family were associated with changes in platelet physiology, the regulation of granule secretion as well as aggregation. Rho GTPases are known key players in cytoskeletal dynamics and therefore serve a critical role in platelets physiology and subsequently vital interactions like haemostasis and inflammation via modulation of platelet activation programs [26]. A down regulation of RhoGDI1 is also seen in patients with hepatocellular carcinoma and is used as a prognostic factor [27]. Through its inhibitory regulation Rho GDI-dissociation inhibitor 1 likely affects the various roles of platelets.

Lastly 14–3-3 gamma is part of the 14–3-3 family of proteins which play a substantial role in signal transduction pathways of eukaryotic cells. Members of the 14–3-3 protein family were previously described in platelets [28]. They play a variety of regulatory roles in various phosphorylation-dependent signalling pathways, among others G-protein signalling [29]. More specifically 14–3-3 proteins interact with several phosphoserine-dependent binding sites in glycoprotein Ib-IX complex (GPIb-IX), the major platelet adhesion receptor, thus affecting and possibly indirectly regulating its interplay with von Willebrand factor (VWF) and mediating platelet activation [30]. One of these interplays is the platelet activation-dependent fibrin formation which can be relevant in haemostasis [31]. Although 14–3-3 zeta is the main part of the 14–3-3 protein family interacting, all six 14–3-3 isotypes are able to bind to GPIb-IX [32]. The interaction of members of the 14–3-3 protein family with GPIb-IX furthermore plays a critical role in enabling the platelet response to low concentrations of thrombin [33].

Thrombocytopenia is one of the most common haematological abnormalities in patients with chronic liver disease, especially in patients with liver cirrhosis. It was long believed that the role of platelets in patients with liver cirrhosis was solely to promote aggregation and form the primary haemostatic plug after adhering to the damaged vessel walls through an interaction with von Willebrand factor [34].

Nowadays, there is growing evidence that platelets, however, play an important role in progression of liver fibrosis through cellular interaction with HSC as well [10]. For example, increasing numbers of platelets as well as platelet-derived chemokine CXCL4 were seen in the immediate vicinity of fibrotic areas in the liver [11]. Platelets were shown to stimulate hepatocyte proliferation in vitro through secretion of hepatic – and insulin like growth factor [35]. Clinical studies have shown that administration of antithrombotic medication such as aspirin leads to a slowing of the development of fibrosis [36]. Interestingly it seems that platelets also may play a role in suppressing HSC activation [37]. Therefore, suppressing the activation from a quiescent state to stellate cells producing extracellular matrix proteins that lead to scar formation [38]. The regulatory role of platelets in liver fibrosis progression is a subject of ongoing research and the cellular mechanisms are still not fully understood.

The changes in the platelet proteome identified in our study might be associated with an altered platelet response, potentially affecting their role in haemostasis or fibrosis progression. Likely by indirectly influencing pathways affecting the platelet response. Such as the previously mentioned 14–3-3 gamma which interacts with the glycoprotein Ib-IX complex which in turn interplays with VWF or Rho GDI 1 which adjusts Rho GTPase activity and affects platelet physiology and subsequent interactions.

In the past, platelet proteomics were successfully performed in a variety of diseases and in many cases identify molecular and functional platelet changes compared to healthy donors. Yet, a more precise assessment of the consequences of platelet proteome alteration is lacking in many cases.

Alcohol consumption exerts a multifaceted impact on platelet function, primarily influencing platelet aggregation and activation pathways [39]. Chronic alcohol exposure has been associated with altered platelet morphology and function, potentially due to ethanol’s direct toxic effects on megakaryocytes and its indirect effects mediated through altered hepatic metabolism [40]. Moreover, alcohol-induced oxidative stress plays a crucial role in modulating the platelet proteome, leading to dysregulation of proteins involved in hemostatic processes [41]. In our study, the direct effects of alcohol on the platelet proteome were minimized as much as possible by adhering to strict exclusion criteria mentioned above, ensuring that observed proteomic changes were predominantly associated with the progression of liver cirrhosis rather than acute alcohol exposure.

As with other platelet proteome studies, this analysis is potentially influenced or limited by several factors. Collection and preparation of samples as well as use of non-antithrombotic medications like anti-depressants might have influenced platelet activation. Standardization of collection and preparation as well as rigid exclusion criteria were applied to minimize such influences. Due to the numerically very low platelet count in patients with advanced cirrhosis, we were able to extract a limited total amount of platelets from these patients. This limited further evaluation of findings in subsequent analysis such as immunoassays or alternative MS-based methods due to our focus of running each sample both in a technical and biological duplicate. This circumstance represents a limitation of our study and should be prioritized in future research endeavors. Furthermore, the sample size, though adequate for initial exploratory analysis, may be inadequate for broad generalizability. This limitation could affect the statistical power and detection of significant differences between groups, as well as the extrapolation of findings to a wider population. Recognizing this constraint is vital for interpreting our results. Lastly, a prospective longitudinal approach would have been more conclusive to show changes throughout disease progression.

In conclusion, we identified several down-regulated proteins thus providing novel information regarding the platelet proteome and its changes in liver cirrhosis patients. Future studies are needed to further clarify the role and significance of the described proteins due to the many pathways and interactions still unclear in platelet regulation. Additionally future research is now needed to determine the clinical significance for haemostatic or immune regulatory function of platelets in cirrhosis.

### Supplementary Information

**Additional file 1. **2D gel of healthy control. Exemplary two-dimensional gel of a healthy control.

**Additional file 2. **2D-gel of less advanced cirrhosis group. Exemplary two-dimensional gel of a patient with less advanced alcohol induced cirrhosis.

**Additional file 3. **2D-gel of advanced cirrhosis group. Exemplary two-dimensional gel of a patient with advanced alcohol induced cirrhosis.

**Additional file 4. **Exemplary two-dimensional gel of a healthy patient. Exemplary two-dimensional gel of a healthy patient used in Fig. 2 to illustrate the statistically significant proteins.

## Data Availability

All data and materials used in this study are available from the corresponding author upon reasonable request.

## Abbreviations

IPG: Immobilized PH-gradient
TCA: Trichloroacetic acid
IEF: Isoelectric focusing
ACD: Citrate-dextrose solution
INR: International normalized ratio
ESLD: End-stage liver disease
vWF: von Willebrand factor
ACS: Acute Coronary Syndrome
Rab-7a: Ras-related protein 7a
RANBP1: Ran-specific GTPase activating protein
Rho GDI 1: Rho-GDP dissociation inhibitor 1
14–3-3γ: 14–3-3 gamma
HSC: Hepatic stellate cells
RAN: Ras-like nuclear
GPIb-IX: Glycoprotein Ib-IX complex
Ras: Rat sarcoma virus
GTP: Guanosine-5′-triphosphate
GDP: Guanosine diphosphate
LC-MS: Liquid chromatography–mass spectrometry
TFA: Trifluoroacetic acid
DTT: Dithiothreitol
IAA: Iodoacetic acid
SDS: Sodium dodecyl sulfate

## References

[1] Violi F (2011). Patients with liver cirrhosis suffer from primary haemostatic defects? Fact or fiction?. J Hepatol.

[2] Giannini EG, Savarino V (2008). Thrombocytopenia in liver disease. Curr Opin Hematol.

[3] Blonski W, Siropaides T, Reddy KR (2007). Coagulopathy in liver disease. Curr Treat Options Gastroenterol.

[4] Tripodi A, Mannucci PM (2011). The coagulopathy of chronic liver disease. N Engl J Med.

[5] Gulley D (2008). Deep vein thrombosis and pulmonary embolism in cirrhosis patients. Dig Dis Sci.

[6] Tripodi A (2011). Hypercoagulability in cirrhosis: causes and consequences. J Thromb Haemost.

[7] Semple JW, Italiano JE, Freedman J (2011). Platelets and the immune continuum. Nat Rev Immunol.

[8] Garraud O (2017). Platelets and immunity: from physiology to pathology. Transfus Clin Biol.

[9] Kisucka J (2006). Platelets and platelet adhesion support angiogenesis while preventing excessive hemorrhage. Proc Natl Acad Sci U S A.

[10] Sullivan BP (2010). Protective and damaging effects of platelets in acute cholestatic liver injury revealed by depletion and inhibition strategies. Toxicol Sci.

[11] Zaldivar MM (2010). CXC chemokine ligand 4 (Cxcl4) is a platelet-derived mediator of experimental liver fibrosis. Hepatology.

[12] Mateos-Cáceres PJ (2010). Different expression of proteins in platelets from aspirin-resistant and aspirin-sensitive patients. Thromb Haemost.

[13] Arias-Salgado EG (2008). Variations in platelet protein associated with arterial thrombosis. Thromb Res.

[14] Liu J, Li J, Deng X (2014). Proteomic analysis of differential protein expression in platelets of septic patients. Mol Biol Rep.

[15] Vélez P, García Á (2015). Platelet proteomics in cardiovascular diseases. Transl Proteom.

[16] Fecher-Trost C (2013). The in vivo TRPV6 protein starts at a non-AUG triplet, decoded as methionine, upstream of canonical initiation at AUG. J Biol Chem.

[17] Keller A (2002). Empirical statistical model to estimate the accuracy of peptide identifications made by MS/MS and database search. Anal Chem.

[18] Nesvizhskii AI (2003). A statistical model for identifying proteins by tandem mass spectrometry. Anal Chem.

[19] Cantalupo G (2001). Rab-interacting lysosomal protein (RILP): the Rab7 effector required for transport to lysosomes. EMBO J.

[20] Walsh TG (2019). Small GTPases in platelet membrane trafficking. Platelets.

[21] Rendu F, Brohard-Bohn B (2001). The platelet release reaction: granules’ constituents, secretion and functions. Platelets.

[22] Guerrier L (2007). Exploring the platelet proteome via combinatorial, hexapeptide ligand libraries. J Proteome Res.

[23] Ren M (1995). Separate domains of the ran GTPase interact with different factors to regulate nuclear protein import and RNA processing. Mol Cell Biol.

[24] Burkhart JM (2012). The first comprehensive and quantitative analysis of human platelet protein composition allows the comparative analysis of structural and functional pathways. Blood.

[25] Garcia-Mata R, Boulter E, Burridge K (2011). The ‘invisible hand’: regulation of RHO GTPases by RHOGDIs. Nat Rev Mol Cell Biol.

[26] Goggs R (2015). Platelet rho GTPases-a focus on novel players, roles and relationships. Biochem J.

[27] Li W (2013). Loss of RhoGDI is a novel independent prognostic factor in hepatocellular carcinoma. Int J Clin Exp Pathol.

[28] Wheeler-Jones CP (1996). Identification of 14-3-3 proteins in human platelets: effects of synthetic peptides on protein kinase C activation. Biochem J.

[29] Gegenbauer K, Nagy Z, Smolenski A (2013). Cyclic nucleotide dependent dephosphorylation of regulator of G-protein signaling 18 in human platelets. PLoS One.

[30] Du X (2007). Signaling and regulation of the platelet glycoprotein Ib-IX-V complex. Curr Opin Hematol.

[31] Cosemans JM (2011). Key role of glycoprotein Ib/V/IX and von Willebrand factor in platelet activation-dependent fibrin formation at low shear flow. Blood.

[32] Chen Y, Ruggeri ZM, Du X (2018). 14-3-3 proteins in platelet biology and glycoprotein Ib-IX signaling. Blood.

[33] Estevez B (2016). Signaling-mediated cooperativity between glycoprotein Ib-IX and protease-activated receptors in thrombin-induced platelet activation. Blood.

[34] Hugenholtz GGC, Porte RJ, Lisman T (2009). The platelet and platelet function testing in liver disease. Clin Liver Dis.

[35] Matsuo R (2008). Platelets strongly induce hepatocyte proliferation with IGF-1 and HGF in vitro. J Surg Res.

[36] Simon TG (2019). Daily aspirin use associated with reduced risk for fibrosis progression in patients with nonalcoholic fatty liver disease. Clin Gastroenterol Hepatol.

[37] Kodama T (2010). Thrombocytopenia exacerbates cholestasis-induced liver fibrosis in mice. Gastroenterology.

[38] Friedman SL (2008). Hepatic stellate cells: protean, multifunctional, and enigmatic cells of the liver. Physiol Rev.

[39] Renaud SC, Ruf JC (1996). Effects of alcohol on platelet functions. Clin Chim Acta.

[40] Levine RF (1986). Effect of ethanol on thrombopoiesis. Br J Haematol.

[41] Ruf JC (2004). Alcohol, wine and platelet function. Biol Res.

