# Supplementary figures and images for: Comparative proteomics reveals different protein expression in platelets in patients with alcoholic liver cirrhosis
Source: Proteome Sci. 2024 Jan 26;22:3. doi: 10.1186/s12953-024-00227-y (PMC10811856; doi:10.1186/s12953-024-00227-y)

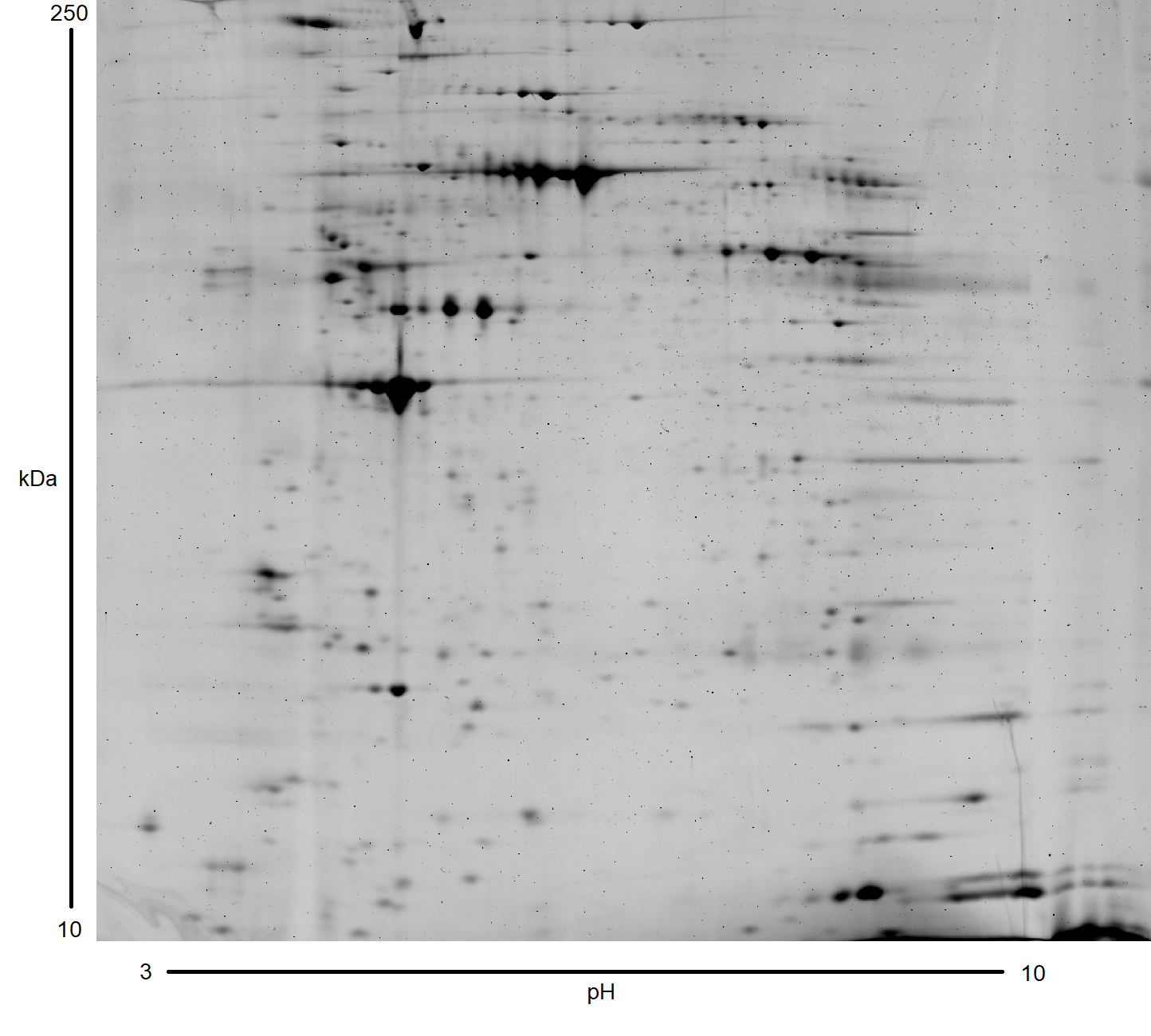

Supplement: Supplementary file 1 — Additional file 1. 2D gel of healthy control. Exemplary two-dimensional gel of a healthy control. [file 12953_2024_227_MOESM1_ESM.tif]

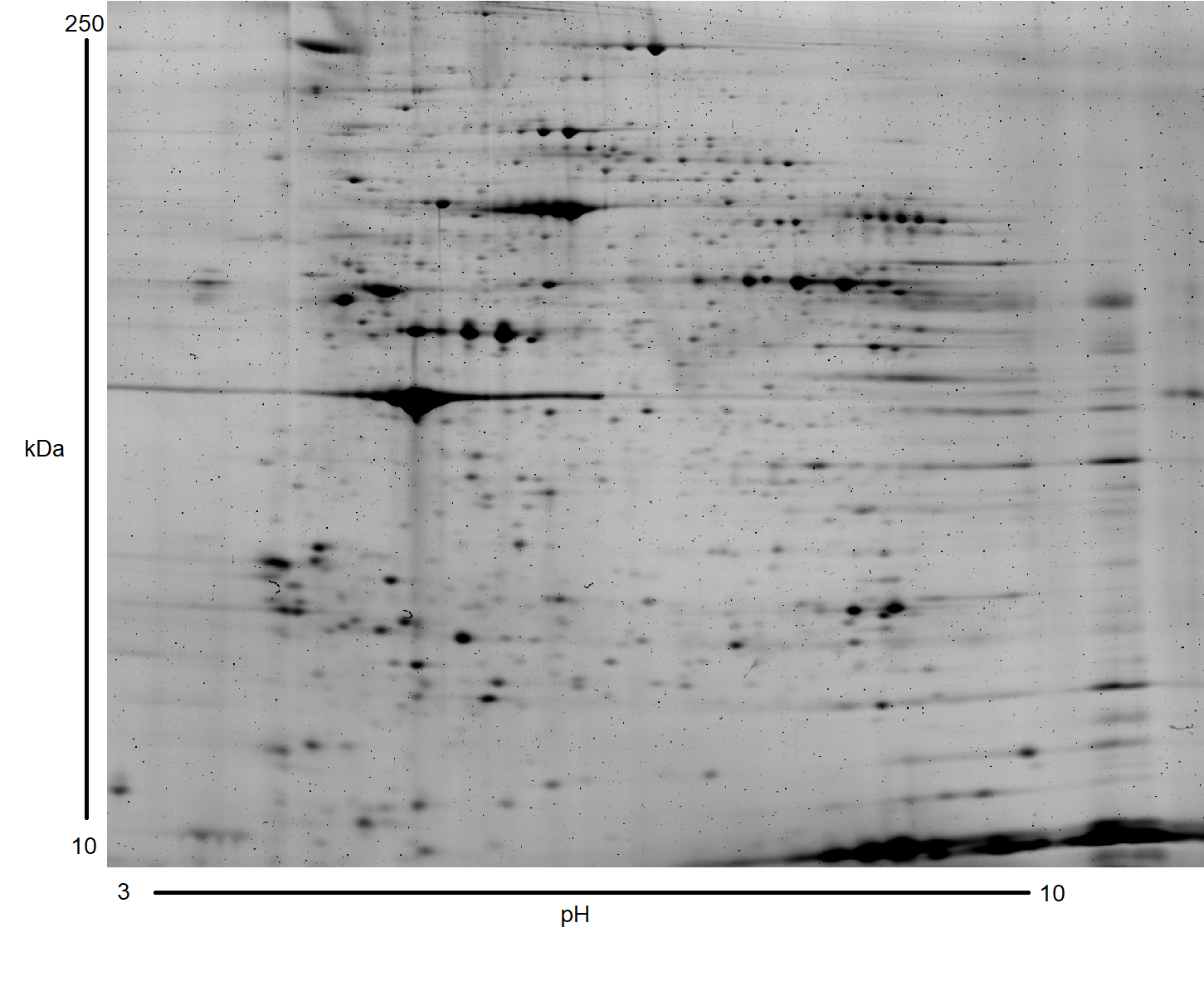

Supplement: Supplementary file 2 — Additional file 2. 2D-gel of less advanced cirrhosis group. Exemplary two-dimensional gel of a patient with less advanced alcohol induced cirrhosis. [file 12953_2024_227_MOESM2_ESM.tif]

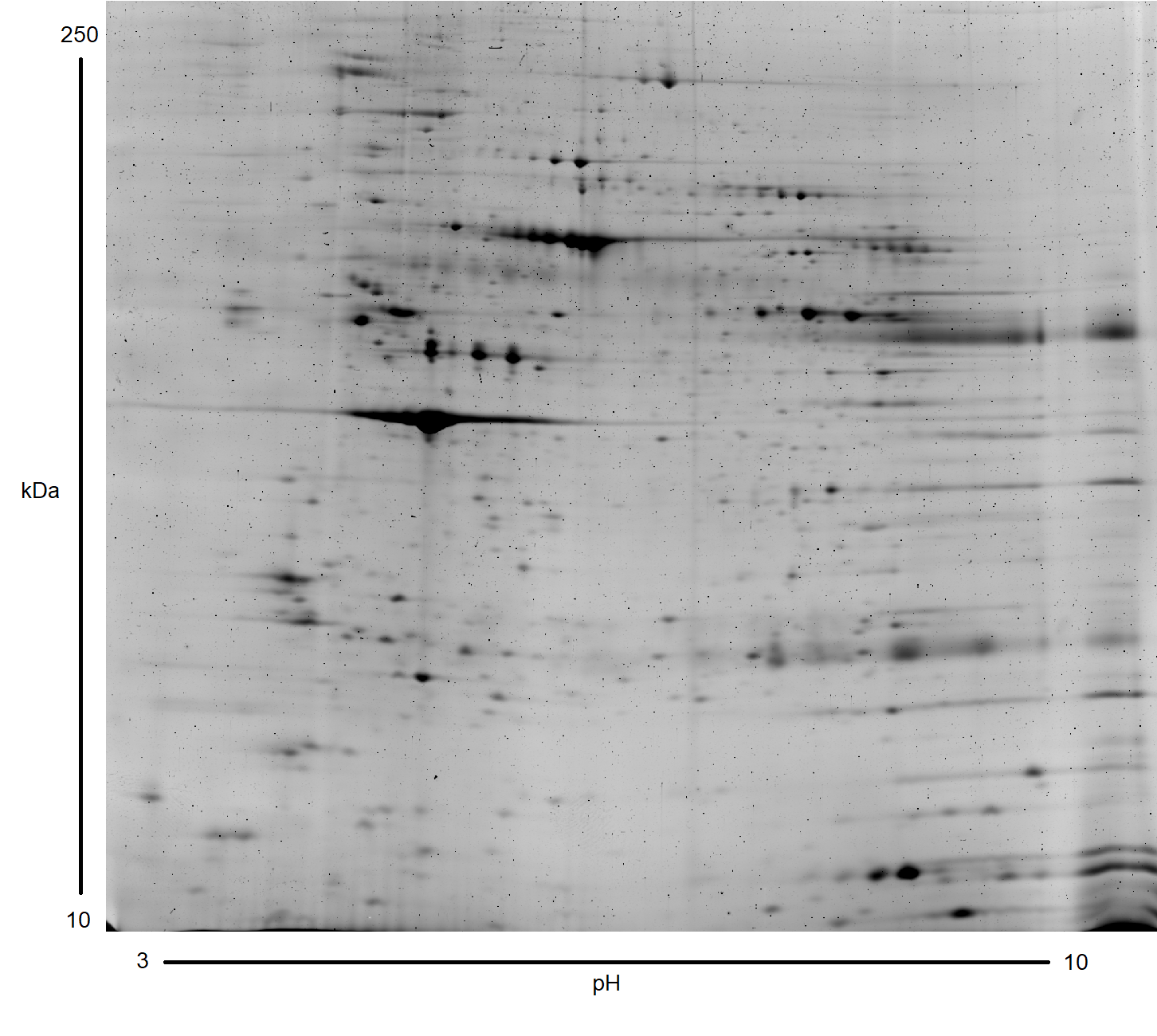

Supplement: Supplementary file 3 — Additional file 3. 2D-gel of advanced cirrhosis group. Exemplary two-dimensional gel of a patient with advanced alcohol induced cirrhosis. [file 12953_2024_227_MOESM3_ESM.tif]

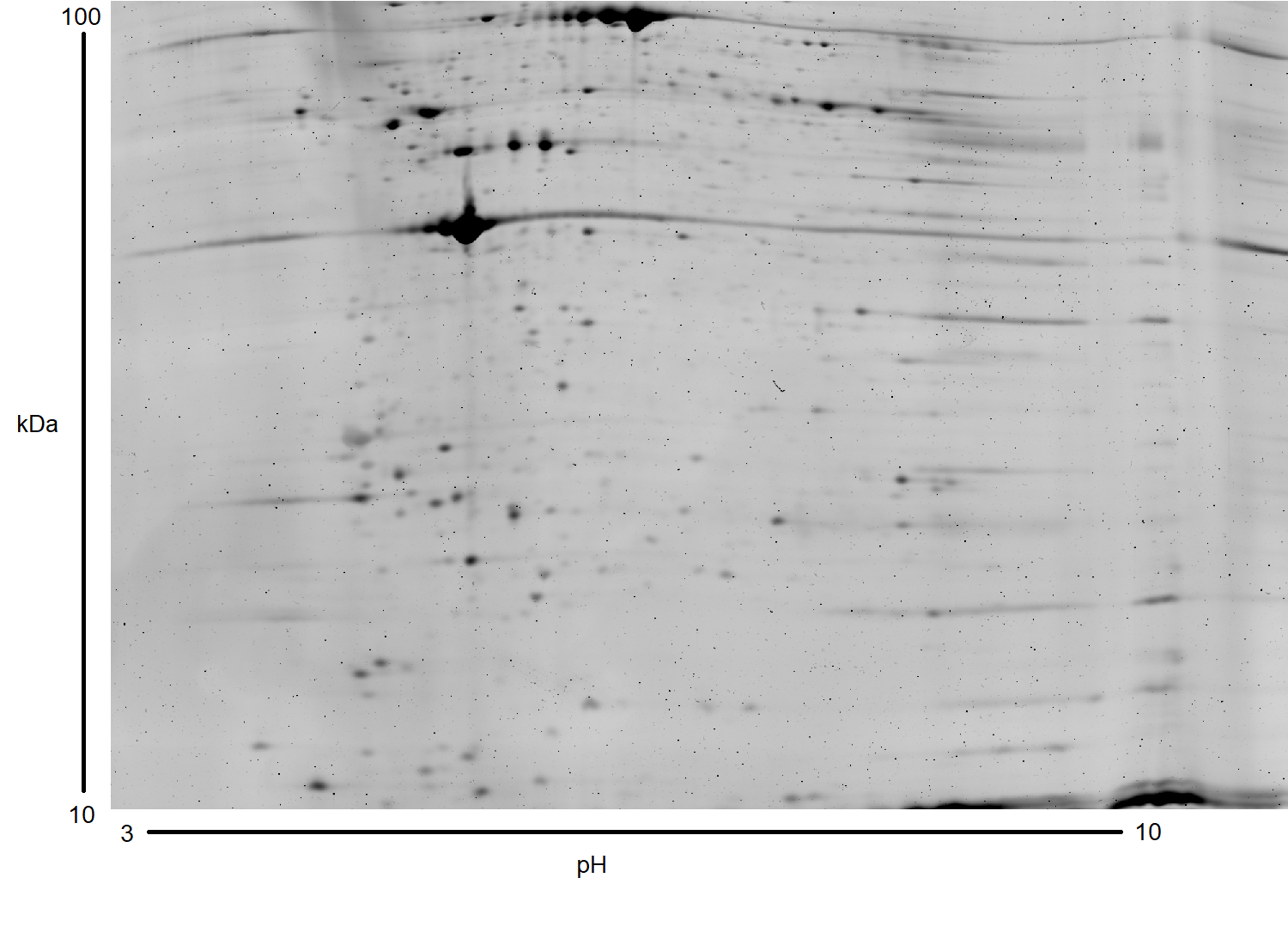

Supplement: Supplementary file 4 — Additional file 4. Exemplary two-dimensional gel of a healthy patient. Exemplary two-dimensional gel of a healthy patient used in Fig. 2 to illustrate the statistically significant proteins. [file 12953_2024_227_MOESM4_ESM.tif]
